# Corticosterone enhances formation of non-fear but not fear memory during infectious illness

**DOI:** 10.3389/fnbeh.2023.1144173

**Published:** 2023-04-06

**Authors:** Alice Hill, Colin Johnston, Isaac Agranoff, Swapnil Gavade, Joanna Spencer-Segal

**Affiliations:** ^1^Michigan Neuroscience Institute, University of Michigan, Ann Arbor, MI, United States; ^2^Division of Metabolism, Endocrinology, and Diabetes, Department of Internal Medicine, University of Michigan, Ann Arbor, MI, United States

**Keywords:** memory, PTSD, glucocorticoids, contextual processing, infectious illness, fear, hippocampus, amygdala

## Abstract

**Introduction:**

Survivors of critical illness are at high risk of developing post-traumatic stress disorder (PTSD) but administration of glucocorticoids during the illness can lower that risk. The mechanism is not known but may involve glucocorticoid modulation of hippocampal- and amygdala-dependent memory formation. In this study, we sought to determine whether glucocorticoids given during an acute illness influence the formation and persistence of fear and non-fear memories from the time of the illness.

**Methods:**

We performed cecal ligation and puncture in male and female mice to induce an acute infectious illness. During the illness, mice were introduced to a neutral object in their home cage and separately underwent contextual fear conditioning. We then tested the persistence of object and fear memories after recovery.

**Results:**

Glucocorticoid treatment enhanced object discrimination but did not alter the expression of contextual fear memory. During context re-exposure, neural activity was elevated in the dentate gyrus irrespective of fear conditioning.

**Conclusions:**

Our results suggest that glucocorticoids given during illness enhance hippocampal-dependent non-fear memory processes. This indicates that PTSD outcomes in critically ill patients may be improved by enhancing non-fear memories from the time of their illness.

## 1. Introduction

More than one in five survivors of critical illness develop post-traumatic stress disorder (PTSD), exhibiting symptoms even up to 8 years after discharge (Kapfhammer et al., 2004; Davydow et al., 2008; Bienvenu et al., 2018; Hatch et al., 2018). These patients also demonstrate significant co-morbidity between PTSD and mood disorders, such as depression and anxiety (Spencer-Segal et al., 2017; Hatch et al., 2018). Glucocorticoids are often administered to critically ill patients, and several studies have found that this exposure to high levels of glucocorticoids during illness is associated with lower levels of PTSD after recovery (Schelling et al., 2001, 2004, 2006; Amos et al., 2014; Sijbrandij et al., 2015). How glucocorticoids influence post-traumatic stress outcomes in critically ill patients is not known. Understanding the mechanisms underlying glucocorticoid modulation of PTSD risk will be crucial for informing critical illness treatment and improving long-term patient outcomes.

Post-traumatic stress disorder symptoms are characterized by pathological memories of the traumatic experience, leading to intrusions, avoidance behavior, and hypervigilance (Schelling et al., 2004; Hauer et al., 2009; Liberzon and Abelson, 2016). Fragmentation of episodic memory, persistent fear memory, and fear generalization are specific memory processes that contribute to the development of PTSD (Liberzon and Abelson, 2016). The neural processes that control episodic, emotional, and fear memories are centered in the amygdala and hippocampus. While the amygdala and ventral hippocampus both play crucial roles in fear memory and its associated emotional arousal (Roozendaal et al., 2009), the dorsal hippocampus is essential for episodic and contextual memory. Together, the hippocampus and amygdala enable accurate recall of the experience, associate this memory with emotion, and allow future discrimination between similar experiences or cues (Pennartz et al., 2011; Yassa and Stark, 2011). The hippocampus and amygdala are both sensitive to glucocorticoids (Meijer et al., 2019), and glucocorticoids are known to modulate fear and non-fear memories. Thus, glucocorticoid treatment during illness might modulate PTSD risk *via* its effect on hippocampal and amygdala processes underlying memory formation.

In this study, we used a mice model of acute infectious illness, cecal ligation and puncture, to study whether glucocorticoids given during an acute illness act on the hippocampus and amygdala to influence the formation and persistence of fear and non-fear memory from the time of the illness. Cecal ligation and puncture (CLP) is a naturalistic sepsis model that we have previously used to study brain outcomes (Singer et al., 2016; Spencer-Segal et al., 2020). CLP induces a real infection with endogenous polymicrobial flora with an illness that lasts several days, during which mice received corticosterone or vehicle injections. The results therefore reflect the interaction between administered glucocorticoids and the inflammatory and endogenous stress response to the illness itself. While previous studies have found that CLP can lead to cognitive dysfunction during illness relative to control mice (Hippensteel et al., 2019; Ge et al., 2023), the long-term persistence of memories from the illness, and their modulation by glucocorticoids, has not been studied. We used adapted versions of the novel object recognition test and contextual fear conditioning in which the habituation or training period occurred during illness and retention was tested 2 weeks later, after complete recovery. We found that glucocorticoids given during illness specifically modulate non-fear memories in survivors, supporting an increased focus on the importance of non-fear memory for trauma outcomes.

## 2. Materials and methods

### 2.1. Animals

Young adult 10–12-week-old C57BL6 male and female mice were obtained from the Jackson Laboratory (*N* = 80 totals, half female). Animals were group housed on a 14:10 light/dark cycle with free access to food and water. All experimental protocols were approved by the University of Michigan Institutional Animal Care and Use Committee and conducted in accordance with the NIH Guide for the Care and Use of Laboratory Animals.

### 2.2. Induction of illness: Cecal ligation and puncture

**FIGURE 1:**
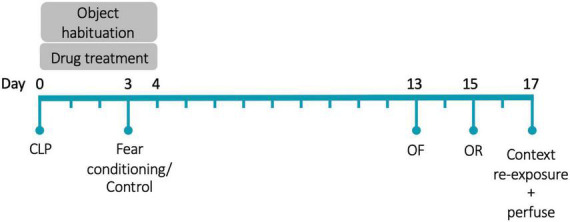
Experimental timeline. CLP, cecal ligation and puncture; OF, open field; OR, object recognition. *N* = 80 animals (20 animals/group, half female).

Animals were anesthetized with isoflurane and were injected with 60 μL of 0.25% bupivacaine at the incision site. Under aseptic conditions, a 5 mm incision was made through the abdominal wall. The cecum was ligated 5 mm from the end with a silk suture and then punctured through-and-through with a 19-gauge needle. The incision was closed with sutures. Surgery was immediately followed with subcutaneous injections of 1 mL saline and 0.1 mL imipenem-cilastatin. The day of surgery is referred to as Day 0. Figure 1 shows the experimental timeline. As we were interested in the effects of glucocorticoid treatment on memory during illness, rather than the effects of illness itself, all animals underwent surgery.

### 2.3. Drug treatment during critical illness

Animals received daily subcutaneous injections of either corticosterone or vehicle on Days 0–4. The corticosterone injections consisted of 0.1 mL of 16 mg/kg corticosterone diluted in sesame oil. The vehicle injections were 0.1 mL of sesame oil. These doses were designed to achieve supraphysiologic corticosterone levels (Mitra and Sapolsky, 2008) and produce a weight-based daily dose analogous to typical glucocorticoid treatment for acutely ill human patients.

### 2.4. Quantification of behavior after recovery from illness

Beginning on Day 13, after full physiologic recovery, animals underwent behavioral testing. Anxiety-like behavior was assessed by measuring exploration in a novel open field, object memory was assessed with a novel object recognition paradigm, and fear memory was assessed with a contextual fear-conditioning paradigm. All behavioral tests, except fear conditioning, were recorded and tracked using Ethovision software (version 15).

#### 2.4.1. Open field test

On Day 13, animals were placed in a novel brightly-lit open field (72 × 72 × 26 cm; 200 lux) and allowed to explore for 5 min.

#### 2.4.2. Novel object recognition

During the concurrent illness period and drug treatment period, animals were habituated to one of two objects (counterbalanced across groups) in their home cage from Day 0 to 4. The objects were small clear cosmetic bottles of similar shapes filled with different colored water; one also had a thick horizontal stripe. On Day 15, after recovery, animals underwent a 5-min object recognition discrimination trial. They were returned to the same open field arena but under low-light conditions (30 lux) and placed equidistant from the two objects: one of which they had been habituated to and the other was novel to them (again, counterbalanced across groups). Investigation of each object was recorded, with the perimeter of the object defined as the object zone.

#### 2.4.3. Contextual fear-conditioning

During the concurrent illness period and drug treatment period (on Day 3), half the animals were subjected to a single footshock training session, consisting of 20 random 1-s low-amplitude (0.45 mA) shocks over 30 min. Repeated low-amplitude shocks induce contextual fear memory lasting at least 24 h (Luyten et al., 2011; Rajbhandari et al., 2018). Olfactory and visual context was provided by 2% acetic acid and a blue-striped curtain. Control animals were placed in the same context but did not receive shocks. On Day 17, after recovery, animals were returned to the fear-conditioned context for 5 min and time spent freezing was recorded using FreezeFrame (ActiMetrics, Wilmette, IL).

### 2.5. Tissue collection

Two hours after testing in the fear-conditioned context on Day 17, all mice were injected with 0.1 mL Nembutal and perfused with 0.1 M phosphate buffer (PB) followed by 4% paraformaldehyde (PFA). Brains were post-fixed with 4% PFA then 30% sucrose and stored at −80°C for future use.

### 2.6. Quantification of neural activity

Coronal brain sections (40 um) were cut serially on a sliding microtome (Leica Biosystems #SM2010R, Buffalo Grove, IL). Sections were rinsed in tris-buffered saline (TBS) and incubated overnight with 1:2000 rabbit primary alpha-mouse c-Fos antibody (SySy #226 003, Goettingen, Germany) in a buffer of bovine serum albumin (BSA), triton, and TBS. After several washes in TBC, sections were incubated with 1:300 biotinylated goat anti-rabbit IgG antibody (Vector Labs #BA-1000, Burlingame, CA) in a buffer of BSA and TBS for 30 min. This was followed by a 30-min incubation in avidin-biotin-peroxidase complex (Vectastain Elite ABC-HRP Kit #PK-6100, Vector Labs, Burlingame, CA). Detection of antibody signal was revealed with 3,3′-diaminobenzidine (DAB) peroxidase substrate (DAB Substrate Kite, Peroxidase with Nickel, Vector Labs #SK-4100, Burlingame, CA).

DAB labeling was visualized using bright-field microscopy. Images were acquired on a Leica DMR-HC microscope (Leica Microsystems), with 5x objective using the same light conditions and acquisition parameters across all animals. A representative section from each animal was selected and matched across animals (dorsal hippocampus: section 74 of 132, ventral hippocampus and basal amygdala: section 86 of 132; Allen Mouse Brain Atlas).^1^ In ImageJ, all images were standardized by subtracting the mean background value from each individual image, selected based on the absence of visible c-Fos immunoreactivity, and then setting to a uniform brightness threshold. A standard-width ribbon tool was used to manually outline principal cell layers of CA1, CA2, and dentate hilus. Area and automated cell count of the region were recorded. Relative c-Fos+ cell density was calculated by dividing the number of c-Fos+ cells by the area. This analysis was repeated for the basal amygdala and for principal cell layers in the dorsal and ventral CA1, CA3, and dentate gyrus. For each of the three brain regions within a single animal, c-Fos+ cell density was quantified in two adjacent sections and averaged. The endpoint for the histology was relative c-Fos+ cell density.

### 2.7. Data analysis

A three-way ANOVA revealed that shock history did not have a significant effect on open field and object recognition behavior, and so the effects of corticosterone and sex on open field behavior were compared using two-way ANOVA. For the object recognition test, several proportions were calculated: the proportion of total object investigation time spent with the familiar object and the proportion of total object investigation time spent with the preferred object. Animals with insufficient total object investigation (less than 3 s) were excluded from this following analysis (*N* = 9 animals). As shock history and sex had no effect on object recognition, the effect of corticosterone on novel object discrimination was compared to the chance value (50%) using an unpaired *t*-test in each of the corticosterone and vehicle groups. As sex also had no effect on freezing behavior, the effects of corticosterone treatment and shock history on freezing behavior were compared with a two-way ANOVA. Statistical analyses were performed using GraphPad Prism 9, with *P* < 0.05 considered significant. Graphs were generated in Prism and show individual data points plus the mean and standard error of the mean (SEM).

For c-Fos immunohistochemistry, a series of multiple linear regression models were generated to predict c-Fos+ cell density in the basal amygdala. Different combinations of the following predictors were included: sex, corticosterone treatment, and history of footshock. These models were compared against a null model and against each other using AICc. For each of the regions of interest in the hippocampus (CA1, CA3, dentate gyrus), a series of linear mixed-effects models were generated to predict c-Fos+ cell density, each including a random intercept to control for individual variation. Different combinations of the following predictors were included: sex, corticosterone treatment, history of footshock, and axis (ventral vs dorsal). The models were compared against a null model and against each other using AIC. These analyses were performed in R using the lme4 and bbmle packages. Graphs were generated in Prism and show individual data points plus the mean and standard error of the mean (SEM). Plots of the predictor effect sizes were generated using the sjPlot package in R.

## 3. Results

We used the gold standard mouse model of systemic infection, cecal ligation and puncture, to study the effects of glucocorticoid treatment during acute infectious illness on the long-term persistence of memories formed during illness (CLP; Buras et al., 2005). A total of 65 of the original 80 (81.25%) mice survived their infectious illness to undergo behavioral testing. Corticosterone treatment did not significantly improve survival; 35 of the 40 (87.5%) corticosterone-treated animals survived, while 30 of the 40 (75%) control animals survived (Fisher’s Exact Test; Relative risk: 0.857, *P* = 0.252).

### 3.1. Corticosterone had no effect on anxiety-like behavior

**FIGURE 2:**
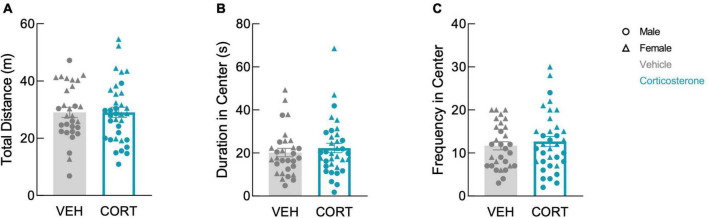
Corticosterone administration did not affect locomotion or anxiety-like behavior in the open field. There was no difference in panel **(A)** total distance traveled, **(B)** total time spent in the brightly-lit center, and **(C)** the number of entries into the center between corticosterone-treated and control animals. In all three measures, females were more active than males (VEH: *N* = 30; CORT: *N* = 35).

We previously showed that mice begin physiologic recovery from CLP within 5 days, with complete recovery of body weight and locomotion within 14 days (Spencer-Segal et al., 2020). Here, we assessed affective behavior in 14-day CLP survivors with an open field test in which mice explored a mildly aversive brightly-lit novel arena. In our prior study, we found that 2-week CLP survivors showed negative affective behavior as compared to a sham operation, and here we asked whether glucocorticoid treatment during illness influences this behavior in CLP survivors. History of footshock did not impact behavior in the open field (total distance: *F*(1,57) = 0.142, *P* = 0.708; time in center: *F*(1,57) = 0.015, *P* = 0.902; frequency in center: *F*(1,57) = 0.142, *P* = 0.707). Therefore, the effects of corticosterone treatment and sex on open field behavior were analyzed using a 2-way ANOVA including both shock and no-shock groups. There was a sex difference in open field behavior: females traveled further (*F*(1,61) = 25.41, *P* < 0.001), spent more time in the center (*F*(1,61) = 7.547, *P* = 0.008), and entered the center more frequently than males (*F*(1,61) = 26.56, *P* < 0.001). We found that corticosterone treatment did not impact exploration in the open field in either sex; corticosterone-treated mice did not differ from vehicle-treated mice in total distance traveled (Figure 2A; *F*(1,61) = 0.004, *P* = 0.951), duration in the brightly-lit center (Figure 2B; *F*(1,61) = 0.496, *P* = 0.484), or frequency in the center (Figure 2C; *F*(1,61) = 0.494, *P* = 0.485). Thus, corticosterone treatment during illness did not affect behavior in the open field after recovery from illness.

### 3.2. Corticosterone facilitated memory of an object from illness

We tested the effect of corticosterone on non-fear memory with a novel object recognition paradigm in which animals were introduced to an object in their home cage during their illness. After recovery, memory of the object from illness was assessed using a discrimination trial in which the animal could freely investigate this familiar object or a novel object inside a familiar arena. Again, history of footshock did not affect behavior in the object recognition test; there was no effect of shock history on total distance (*F*(1,57) = 0.015, *P* = 0.903), total object investigation (*F*(1,57) = 0.352, *P* = 0.555), or proportion of time spent with familiar object (*F*(1,48) = 0.007, *P* = 0.932). The effects of corticosterone and sex on total distance traveled were therefore analyzed using 2-way ANOVA including both shock and no shock groups. As there was also no effect of sex on total object investigation (*F*(1,57) = 0.220, *P* = 0.641) or proportion of time spent with familiar object (*F*(1,48) = 0.248, *P* = 0.621), the effect of corticosterone on object recognition was analyzed using an unpaired *t*-test including both males and females.

**FIGURE 3:**
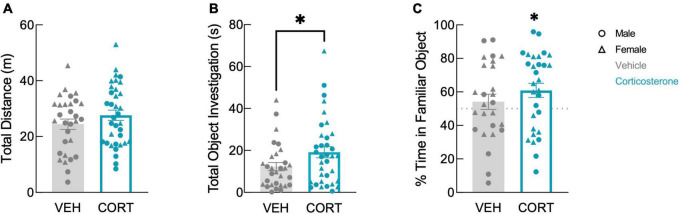
Corticosterone-treated mice showed improved object recognition. Corticosterone-treated animals exhibited **(A)** similar levels of overall activity as control animals but **(B)** spent more time investigating the objects. Corticosterone-treated animals demonstrated **(C)** a preference for the familiar object over the novel object (VEH: *N* = 30; CORT: *N* = 35). *P < 0.05.

In the discrimination trial, corticosterone-treated mice showed no difference in locomotion in the arena (Figure 3A; *F*(1,61) = 1.557, *P* = 0.217). In contrast, corticosterone did influence overall object investigation: corticosterone-treated animals spent more total time investigating the objects than vehicle-treated animals (Figure 3B; *t*(63) = 2.011, *P* = 0.049). Corticosterone-treated mice also tended to spend more time with the familiar object than did those treated with vehicle (*t*(63) = 1.982, *P* = 0.052), but there was no effect of corticosterone treatment on time spent investigating the novel object (*t*(63) = 1.138, *P* = 0.260). This suggests that animals treated with corticosterone selectively investigated the familiar object over the novel object. Indeed, corticosterone-treated animals exhibited a significant preference for the familiar object (*t*(29) = 2.599, *P* = 0.015), while vehicle-treated animals had no preference (Figure 3C; *t*(25) = 0.897, *P* = 0.378). The discrimination between the novel and familiar object in corticosterone-treated mice suggests that corticosterone-treated mice recalled a long-term memory of the object last seen 11 days prior, while vehicle-treated animals did not.

### 3.3. Corticosterone did not affect fear memory from illness

To assess the effects of corticosterone on fear memory persistence after illness, we used a contextual-fear conditioning paradigm in which animals underwent a single training trial during the illness period. Two weeks later, after recovery, animals were re-exposed to the conditioned context in a single testing session and freezing behavior was recorded to measure fear memory.

**FIGURE 4:**
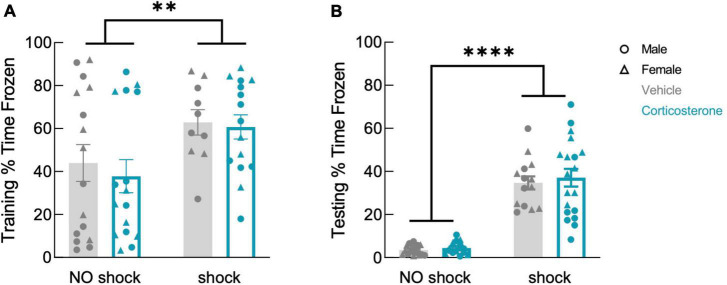
History of footshock, but not corticosterone administration, affected contextual fear memory. Animals that had experienced shocks from more than control animals in both **(A)** training and **(B)** testing trials, while corticosterone-treated and vehicle-treated animals froze at equal rates (training: no shock *N* = 32, shock *N* = 25; testing: no shock *N* = 32, shock *N* = 33). ^**^P < 0.01; ^****^P < 0.0001.

Corticosterone treatment did not affect freezing during training (Figure 4A; *F*(1,53) = 0.297, *P* = 0.588), which was higher in the shock than in the no-shock group (*F*(1,53) = 7.633, *P* = 0.008). During retention testing, fear-conditioned mice showed increased freezing in the conditioned context (Figure 4B; *F*(1,61) = 128.9, *P* < 0.0001), demonstrating that all animals formed a lasting fear memory, with no effect of corticosterone (*F*(1,61) = 0.350, *P* = 0.557). Corticosterone therefore had no effect on fear memory in this assay, in contrast to its facilitation of object memory.

### 3.4. Corticosterone treatment enhanced neural activity associated with a familiar context

**FIGURE 5:**
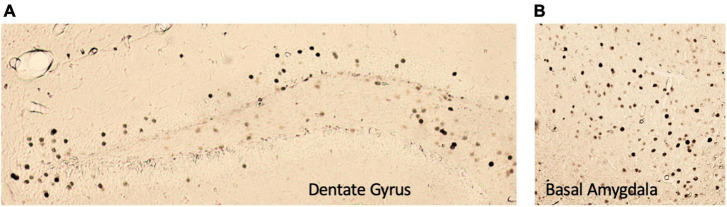
Representative images of c-Fos+ immunoreactivity in the **(A)** dentate gyrus and **(B)** basal amygdala. All images were taken at 5× magnification.

**FIGURE 6:**
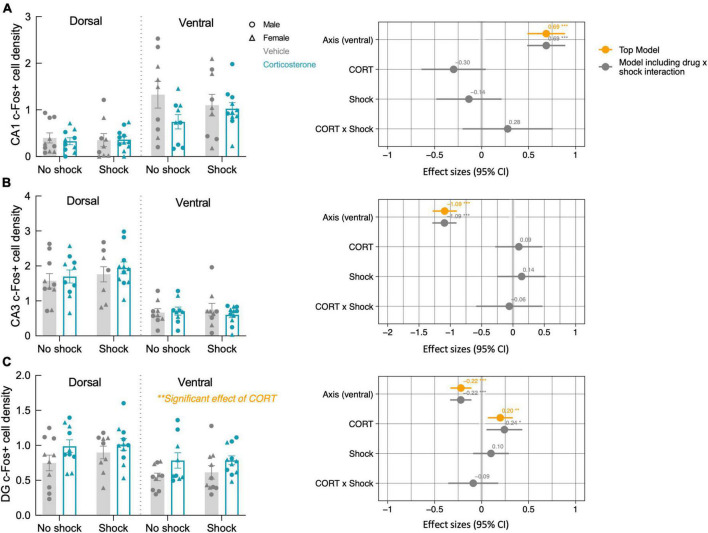
Corticosterone treatment increased neural activity in the dentate gyrus during context re-exposure. The density of c-Fos+ cells in the **(A)** CA1 and **(B)** CA3 were not affected by history of footshock or corticosterone administration. **(C)** In the dentate gyrus (DG), c-Fos+ cell density was greater in corticosterone-treated animals than control animals. The coefficient plot on the right of each panel shows the effect sizes of the predictors with a 95% confidence interval. The orange points represent the effect sizes of the predictors in the top model, while the gray represents the predictor effect sizes of the model that includes the CORT × Shock interaction term. Only in the dentate gyrus does corticosterone treatment appear as a predictor in the top model, with a significant positive effect on neural activity. *P < 0.05; ^**^P < 0.01; ^***^P < 0.0001.

After the context re-exposure during the testing trial, mice were euthanized for quantification of neural activity in the hippocampus and basal amygdala using c-Fos immunohistochemistry as a proxy for neuronal activation in those brain regions (Krukoff, 1999; Figure 5). Significant predictors of c-Fos+ cell density were identified *via* linear regression and model comparison. In the CA1 and CA3, there was no significant contribution of corticosterone treatment and shock (Figures 6A, B). The top model for both regions included only axis as a predictor, indicating that neural activity was higher in the ventral area (Table 1). The models that included corticosterone treatment and shock as predictors did not fit the data as well, and the effect sizes for these predictors were not significant. In the dentate gyrus, animals treated with corticosterone had greater c-Fos+ cell density compared to animals treated with vehicle (Figure 6C; *P* = 0.002), suggesting greater activation of the dentate gyrus during context re-exposure in the corticosterone-treated mice. The top model for the dentate gyrus included corticosterone as a predictor and the effect size was significant (Table 1). In all hippocampal regions, there was no interaction between the effect of corticosterone treatment and the effect of history of footshock on neural activity; the models that included an interaction between corticosterone treatment and shock fit the data poorly and the effect size of the interaction term was not significant (Table 1).

**TABLE 1:** Model selection for the effect of axis (ventral), history of footshock (shock), corticosterone treatment (CORT), and sex on c-Fos+ cell density in the hippocampus.

Region	Top models (plus interaction model)	AIC	δAIC	df	Weight
CA1	**cFos_density ∼ axis + (1| ID)**	**121.7**	**0.0**	**4**	**69.9%**
	cFos_density ∼ axis + CORT + (1| ID)	124.4	2.7	5	18.1%
	cFos_density ∼ axis + shock + (1| ID)	126.1	4.4	5	7.9%
	cFos_density ∼ axis + CORT + shock + (1| ID)	128.8	7.1	6	2.0%
	cFos_density ∼ axis × shock + CORT + (1| ID)	130.1	8.4	7	1.0%
	…				
	cFos_density ∼ axis + CORT × shock + (1| ID)	130.5	8.8	7	0.9%
CA3	**cFos_density ∼ axis + (1| ID)**	**123.7**	**0.0**	**4**	**72.6%**
	cFos_density ∼ axis + shock + (1| ID)	127.3	3.5	5	12.4%
	cFos_density ∼ axis + CORT + (1| ID)	127.7	4.0	5	9.9%
	cFos_density ∼ axis + CORT + shock + (1| ID)	131.3	7.5	6	1.7%
	cFos_density ∼ axis + CORT + shock + sex + (1| ID)	131.4	7.7	7	1.6%
	…				
	cFos_density ∼ axis + CORT × shock + (1| ID)	134.1	10.3	7	0.4%
Dentate gyrus	**cFos_density ∼ axis + CORT + (1| ID)**	**39.1**	**0.0**	**5**	**71.3%**
	cFos_density ∼ axis + (1| ID)	42.0	2.8	4	17.4%
	cFos_density ∼ axis + CORT + shock + (1| ID)	44.1	4.9	6	6.0%
	cFos_density ∼ axis + shock + (1| ID)	46.8	7.6	5	1.6%
	cFos_density ∼ axis + CORT + shock + sex + (1| ID)	47.7	8.5	7	1.0%
	…				
	cFos_density ∼ axis + CORT × shock + (1| ID)	47.8	8.7	7	0.9%

Linear mixed models using different combinations of predictors and controlling for individual variation (ID) were fit for each of the three hippocampal brain regions separately. For each region, the top five models are displayed, in addition to the interaction model (which reliably fit the data poorly). Lower AIC values indicate better fitting models, with the top model in each region indicated by bold text. Only in the dentate gyrus did the top model include corticosterone treatment as a significant predictor of neural activity.

**FIGURE 7:**
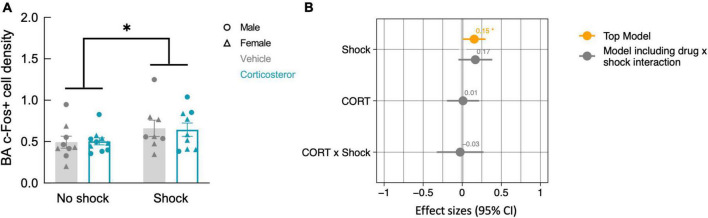
History of footshock, but not corticosterone treatment, increased neural activity in the basal amygdala during context re-exposure. **(A)** The density of c-Fos+ cells was greater in animals previously exposed to footshocks than in control animals. **(B)** The top linear regression model included only history of footshock as a predictor, with a significant positive effect size. The model including an interaction term between corticosterone treatment and shock was a worse fit and included no meaningful effect of the interaction or of each independent predictor. *P < 0.05.

While fear conditioning (shock vs. no shock) did not affect c-Fos+ cell density in the hippocampus, it did so in the basal amygdala, where fear conditioned mice did show increased c-Fos+ cell density (Figure 7A; *P* = 0.038). The top model included only history of footshock as a predictor, and the effect size was significant (Table 2). There was no effect of corticosterone treatment on c-Fos+ in the amygdala and again, there was no significant interaction between the effect of corticosterone treatment and history of footshock (Figure 7B). This is indicated by the poorly fitting interaction model, in which the effect size of the interaction term was not significant (Table 2). In conclusion, c-Fos+ immunohistochemistry demonstrated increased neuronal activation in the dentate gyrus during context re-exposure in corticosterone-treated mice, independent of fear conditioning.

**TABLE 2:** Model selection for the effect of history of footshock (shock), corticosterone treatment (CORT), and sex on c-Fos+ cell density in the basal amygdala.

Top five models (plus interaction model)	AICc	δAICc	df	Weight
**cFos_density ∼ shock**	**−4.6**	**0.0**	**3**	**46%**
cFos_density ∼ 1 (null model)	−2.3	2.2	2	15.1%
cFos_density ∼ CORT **+** shock	−2.0	2.5	4	12.9%
cFos_density ∼ CORT **+** shock **+** sex	−1.7	2.8	5	11.1%
cFos_density ∼ CORT	0.1	4.6	3	4.6%
…				
cFos_density ∼ CORT × shock	0.7	5.2	5	3.4%

Linear models using different combinations of predictors were compared using AICc, due to small sample size. The poorly fitting interaction model is also included for reference. Lower AICc values indicate better fitting models. The top model (in bold) includes history of shock as a significant positive predictor of neural activity in the basal amygdala.

## 4. Discussion

We found that glucocorticoid treatment during illness impacted the persistence of non-fear memory but not fear memory after recovery. Administration of corticosterone during CLP-induced illness facilitated long-term memory of an object from the illness period. In contrast, neither affective behavior nor the expression of contextual fear memory was affected by corticosterone treatment. Corticosterone treatment was also associated with elevated neural activity in the dentate gyrus during context re-exposure independent from fear conditioning. This increase in dentate gyrus neural activation during context re-exposure suggests that corticosterone promoted memory of the context, rather than fear memory *per se*. Taken together, our data suggest that corticosterone given during illness primarily impacts hippocampal-dependent non-fear memory processes. In the context of the repeated finding, in humans, that glucocorticoid treatment during illness decreases the risk of PTSD in survivors, our data suggest the intriguing possibility that glucocorticoids treatment prevent PTSD by improving non-fear memories from the illness.

As mice are assumed to prefer novelty, we were surprised to see that the animals spent more time with the familiar than the novel object during the object discrimination test. While unusual, the apparent behavioral discrimination still suggests intact memory of the familiar object. As all animals underwent multiple types of memory training, prior experiences may influence behavioral outcomes; however, contextual fear conditioning does not seem to have had a measurable effect on behavior in the open field or object recognition test, as animals with a history of footshock did not behave significantly differently from control (no shock) animals in those tests.

There are several possible explanations for the unusual results in the object recognition test. The preference for the familiar object could be due to a positive emotional valence attached to the object during illness. Alternatively, it could represent novelty aversion, as novelty preference is distinct from memory *per se*. Indeed, there is evidence that rats can retain long-term object recognition while demonstrating a preference for the familiar object and that the perirhinal cortex may be involved in the switch in preference from novelty to familiarity (Mumby et al., 2002). Another explanation could be that the discrimination trial was run in a familiar arena while mice had previously seen the object in their home cage, leading to recognition of an object-context mismatch during the discrimination trial (Sheldon, 1969; Thakral et al., 2015). Finally, we used a novel object recognition paradigm with a protracted time frame, involving 4 days of object habituation followed by a delay of almost 2 weeks prior to testing. Multiple days of exposure to an object have previously been used for object recognition in rodents and indeed can increase the durability of apparent object memory to several weeks, supporting the feasibility of our task (Mumby et al., 2002). The shift in preference to the familiar stimulus in our study could be explained by the long delay between initial stimulus presentation and the discrimination task, as longer delays shifted preference from the novel to the familiar stimulus in human infants and adults (Bahrick, 1995; Richmond et al., 2007). The neural basis for this shift in preference is not known but may involve a requirement for updating and reconsolidating a remote memory of the familiar object (Ennaceur, 2010).

Contextual fear memory depends on both the hippocampus and the amygdala. The amygdala, particularly the basal amygdala, is typically associated with the emotional aspect of the memory, while the hippocampus is thought to pair the fear memory to the context (Phillips and LeDoux, 1992; Kim and Cho, 2020). Previous studies investigating the role of glucocorticoids in the hippocampus and amygdala on fear memory persistence have produced mixed results. Targeted activation of glucocorticoid receptors in the basal amygdala enhanced fear memory in an inhibitory avoidance test (Roozendaal and McGaugh, 1997) and targeted inhibition led to impaired contextual fear conditioning (Donley et al., 2005), suggesting that glucocorticoids acting in the amygdala enhance fear memory formation. In the latter study, impaired performance was also caused by inhibition of receptors in the ventral hippocampus, but not dorsal hippocampus. In contrast, systemic administration of high doses of glucocorticoids following acute stress impaired contextual fear memory (Cohen et al., 2008). This literature suggests that the dorsal hippocampus and ventral hippocampus/amygdala might play opposing roles in fear memory formation. How these effects might be integrated to produce an effect of systemic glucocorticoids on fear memory formation in the context of an acute illness has not previously been tested. While our methods did not enable us to isolate the specific effects of glucocorticoids in each region, the greater similarity to clinical glucocorticoid administration provides greater translational relevance.

We found that corticosterone did not affect contextual fear memory after conditioning during CLP-induced illness. The relatively high freezing across all groups during the training session is likely a result of reduced locomotion induced by CLP, as seen previously (Spencer-Segal et al., 2020); however, animals receiving shocks still froze more than control animals. By the testing session, control animals exhibited negligible freezing, as would be expected from animals not exposed to shocks and indicating a complete recovery from CLP. In contrast, all animals exposed to shocks showed good memory retention during the testing session, which was not altered by corticosterone treatment. It is possible that in our study we observed a “ceiling effect” considering the high efficacy of our fear conditioning regiment in both corticosterone-treated and untreated groups. This potential “ceiling effect” could also be a product of the high endogenous catecholamine and glucocorticoid levels as well as the inflammatory response to the illness (Gupta and Guleria, 2022). The results suggest that, in the setting of this neuroendocrine and inflammatory background, supplemental glucocorticoids do not have a significant modulatory effect on the development of fear memory. The findings are consistent with several clinical studies that find that the preventative effect of glucocorticoids against PTSD after critical illness is not associated with reduced numbers or types of fear memory (Schelling et al., 2001, 2004; Weis et al., 2006). It is also possible that the effects of glucocorticoids on fear memory were not captured with our strong conditioning paradigm. Results could differ depending on the strength and type of conditioning (Maren, 2008). Other studies have found that PTSD is marked by abnormal extinction recall and fear renewal (Garfinkel et al., 2014); perhaps if we had tested the animals for extinction, extinction recall, and renewal of the fear memory, we would have seen an effect of glucocorticoid history on behavior and amygdala activity.

C-Fos cell density analysis revealed greater neural activity in the basal amygdala in the fear-conditioned group, supporting the role of amygdala in fear memory recall and consistent with the lack of effect of corticosterone on freezing behavior during the testing trial. In contrast, animals treated with corticosterone exhibited greater neural activity in the dentate gyrus during context re-exposure, irrespective of fear conditioning. As granule cells in the dentate gyrus support context discrimination and memory recall (Hainmueller and Bartos, 2020), this potentially indicates improved recognition of the testing context in corticosterone-treated mice, consistent with the above improvement in non-fear memory from the illness period. Consistent with a possible role for the dentate gyrus in the lasting effects of glucocorticoid treatment, a previous study showed that hydrocortisone given to stress-exposed animals caused lasting increases in dendritic growth and spine density in dentate granule cells (Zohar et al., 2011). The dentate gyrus has previously been implicated in the stimulus overgeneralization and subsequent inappropriate emotional responses seen in disorders such as PTSD (reviewed in Kheirbek et al., 2012). Our findings suggest that glucocorticoids may act at the dentate to improve recall of non-fear memories from illness and decrease PTSD risk.

Other brain regions previously implicated in stress-associated memories include the medial prefrontal cortex (mPFC). While the hippocampus encodes memories of the context, the PFC is responsible for associating these memories with emotional responses (Euston et al., 2012). The two regions demonstrate strong connectivity and are functionally linked (Cenquizca and Swanson, 2007; Herweg et al., 2016). Like the hippocampus, there is high glucocorticoid sensitivity in the mPFC and, in fact, expression of contextual fear conditioning depends upon activation of glucocorticoid receptors in the mPFC (Reis et al., 2016). In addition, diminished mPFC signaling has been repeatedly associated with PTSD (Etkin and Wager, 2007). In the future, it would be interesting to investigate the effect of glucocorticoid treatment on mPFC activity during recall of fear and non-fear memories from illness.

Taken together, our findings suggest that corticosterone treatment during illness improves non-fear memory from the illness period without affecting fear memory. Accurate recall of the ICU experience (rather than delusional or emotional memories) has been proposed to protect against PTSD in survivors (Jones et al., 2001); this is the presumed mechanism for clinical interventions such as ICU diaries (Jones et al., 2010; Garrouste-Orgeas et al., 2012, 2019; Wang et al., 2020). Our findings suggest that glucocorticoids may act *via* this mechanism during illness and suggest that continued focus on enhancing non-fear memories from the illness period may be valuable PTSD prevention strategies in critically ill patients and survivors.

## Data availability statement

The raw data supporting the conclusions of this article will be made available by the authors, without undue reservation.

## Ethics statement

The animal study was reviewed and approved by the University of Michigan Institutional Animal Care and Use Committee.

## Author contributions

JS-S and AH applied for grants, designed the experiment, and wrote the manuscript. AH created the figures. AH, CJ, and IA performed the experiments. SG contributed to the immunohistochemistry data analysis. All authors read and agreed to the published version of the manuscript.

## Footnotes

1
https://mouse.brain-map.org/static/atlas
